# Imagined Intergroup Physical Contact Improves Attitudes Toward Immigrants

**DOI:** 10.3389/fpsyg.2018.01685

**Published:** 2018-09-18

**Authors:** Soraya E. Shamloo, Andrea Carnaghi, Valentina Piccoli, Michele Grassi, Mauro Bianchi

**Affiliations:** ^1^Department of Life Sciences, University of Trieste, Trieste, Italy; ^2^HEI-Lab, University Lusófona/ISCTE—Lisbon University Institute, Lisbon, Portugal

**Keywords:** touch, physical contact, imagined contact, prejudice, immigrants, intergroup bias, implicit attitudes

## Abstract

In this set of research, we investigated the effects of intergroup physical contact on intergroup attitudes by relying on indirect contact strategies, namely the imagined contact paradigm. We implemented the imagined contact paradigm by leading participants to shape the mental imagery upon pictorial information. Specifically, in Study 1 participants saw a picture of a white hand touching a black hand [i.e., intergroup physical contact condition (InterPC)] or a picture of an outdoor scene (i.e., control condition), and were asked to imagine being either the toucher or in the outdoor scene, respectively. Results demonstrated that InterPC compared to control condition reduced intergroup bias. In Study 2 we compared the InterPC condition to a condition in which participants saw a white hand touching another white hand [i.e., intragroup physical contact (IntraPC)], and imagined to be the toucher. Again, we found that participants in the InterPC condition showed reduced intergroup bias compared to the IntraPC. Study 3 replicated results of Studies 1 and 2 by using an implicit measure of prejudice. Also, Study 3 further showed that asking participants to merely look at the picture of a white hand touching a black hand, without imagining being the toucher was not effective in reducing implicit prejudice. Results were discussed with respect to the literature on physical contact and prejudice reduction processes.

## Introduction

Touch is among human senses the first to develop and it is mainly through touch that infants interact with others (Montagu, 1971; Field, 2001; Hertenstein et al., 2006b). Though language then becomes a central source of communication, touch still remains an important communicative tool throughout adulthood (Hertenstein et al., 2009). Touch positively impacts on impression formation, and promotes prosocial behavior at interpersonal level (see Gallace and Spence, 2010). Importantly, physical contact with an outgroup member constitutes the ground for generalizing the positive encounter to the outgroup as a whole, thus reducing outgroup prejudice (Seger et al., 2014). Some factors may undermine the achievable positive outcomes of physical contact in intergroup settings. Cultural norms regulating social interactions rule and, at least in certain cases, constrain the use of physical contact (Field, 2001). In some circumstances, touch can be also perceived as a vector of pathogen transmission, as in the case of intergroup physical contact (Neuberg et al., 2011; Golec de Zavala et al., 2014). Hence, cultural norms and self-protecting motivations can boost the tendency to avoid physical contact in social contexts in general, and intergroup contexts in particular. As a consequence, the possibility of using physical contact *per se* as a way to improve the quality of interpersonal interaction and possibly to reduce prejudice is strongly challenged.

The specific contribution of this work is to start posing the question of whether indirect forms of physical contact, which do not require face-to-face intergroup interactions, are effective in achieving prejudice reduction.

The current set of studies intends to bridge two strands of literatures that have developed independently so far, namely the research on physical contact and the studies on indirect forms of intergroup contact. With respect to research on physical contact, given the very limited amount of research that has addressed whether touch in intergroup settings improves intergroup attitudes, the current set of studies intends to corroborate evidence on the positive effect of intergroup touch, by exploring its effectiveness beyond the direct contact. With few exceptions (Choma et al., 2014; Hodson et al., 2015), research on indirect intergroup contact has mainly focused on positive and generic interactions with outgroup members. Hence it is still not clear whether specific forms of indirect intergroup contact, as those represented by physical contact, are also effective in improving intergroup relations. The current set of studies intends to gather evidence on this issue by analyzing whether a specific form of indirect contact, namely a positive intergroup physical encounter, may work as a basis for improving intergroup relations.

### Physical Contact: From Interpersonal to Intergroup Contexts

Empirical evidence on physical contact has prevalently addressed the effects of touch at interpersonal level. Interpersonal touch exerts a strong impact on human’s well-being (Jakubiak and Feeney, 2016), as demonstrated by decreased cortisol levels, an increase in oxytocin levels (Ditzen et al., 2007; Holt-Lunstad et al., 2008), and reduced feelings of pain in patients with chronic pain conditions (see Field et al., 2007). Touch also influences interpersonal interactions (see Gallace and Spence, 2010). A casual touch between strangers improves the evaluation of the toucher (Erceau and Guéguen, 2007). Moreover, willingness to engage in prosocial behaviors is enhanced when touch occurs between the potential helper and who is in need (Guéguen and Fischer-lokou, 2003). Touch also helps creating “communal sharing” relationships in which the other comes to be seen like oneself. Indeed, touch affects human relations, as touch makes sharing resources become part of the relationship’s modus operandi, like in the caregiver-child relationship (Fiske, 1992, 2004).

Notwithstanding the contribution of the above-mentioned studies to the understanding of how touch positively impacts on interpersonal contexts, only few studies have addressed whether these positive effects may also be extended to outgroup members, and possibly to attitudes toward the outgroup as a whole. As far as interethnic touch is concerned, the effect of touch has been investigated either by analyzing physiological reactions and attitudes in a *hic et nunc* intergroup interaction (Rankin and Campbell, 1955; Vrana and Rollock, 1998; Seger et al., 2014), or by addressing the consequences of a contextual interethnic touch on attitudes toward the outgroup as a whole (Seger et al., 2014).

Research assessing physiological reactivity in a physical interethnic encounter has typically relied on experimental procedures in which European–American students were touched either by an African–American or a European–American experimenter. This line of research has produced mixed findings. Studies completed in the 1950 and 1960s showed that interethnic touch triggered high levels of physiological reactivity (i.e., high levels of skin conductance) in European–American students. More recent research has instead shown that being touched by an European–American or an African–American elicits a similar physiological reactivity in European–American participants, both in terms of skin conductance and facial expression (i.e., zygomatic and corrugator, Vrana and Rollock, 1998). The discrepancy between the results of these two studies might be attributed to historical and cultural changes both in terms of diversity in the University campus, that contextually increases the familiarity with African–Americans (National Center for Education Statistics [NCES], 2016), and also in terms of less general negative public opinion toward African–Americans compared to the past (Firebaugh and Davis, 1988). Corroborating this conjecture, recent findings have shown that being touched by an African–American triggers, at least nowadays, a positive evaluation of the toucher (Seger et al., 2014). Specifically, Seger et al. (2014) reported that European–American participants showed a positive evaluation of an African–American experimenter, both when they only interacted with the experimenter as well as when the interaction was further qualified by an intergroup touch. Importantly, although the positive evaluation of the experimenter was found both when interacting and when also physically interacting with the outgroup member, the positive intergroup encounter generalized to the outgroup representation, thus weakening outgroup prejudice, especially when participants were touched by the African–American experimenter. This study demonstrates that when a positive intergroup contact has been established, physical contact facilitates the generalization of the positive experience of the intergroup encounter to the outgroup as a whole.

Although the authors claimed that being touched by anyone, including an individual who shared the participant’s race (Seger et al., 2014), rather than an outgroup member, was highly unlikely to produce comparable effects on outgroup prejudice, this claim has not been directly addressed yet. Indeed, whether it is a touch *per se*, which could constitute a positive experience, or an intergroup touch specifically the generative mechanism of prejudice reduction, still remains an open issue.

The current set of studies aimed at gathering additional evidence on the positive relationship between intergroup touch and intergroup attitudes, given the limited amount of research on this issue. Furthermore, we aim at ascertaining whether a touch with an outgroup member rather than an individual likely pertaining to the ingroup, can account for prejudice reduction, as previous research has failed to clarify this point. Importantly, these aims are fulfilled by investigating physical contact within a novel context, namely the imagined physical contact.

### Imagined Physical Contact and Prejudice

Physical contact has numerous effects on a wide range of situations including the possibility of ameliorating intergroup attitudes. Unfortunately, establishing physical contact in general, and in intergroup contexts in particular is not always straightforward, for different albeit related reasons. First, physical encounters are subject to cultural variation, with some cultures promoting physical contact while other cultures discourage physical interactions (e.g., Remland et al., 1995). This cultural difference limits the possibility of relying on physical contact to those cultures in which this form of interaction is not at odds with cultural prescriptions. Second, when interacting with outgroup rather than ingroup members, physical distance is typically enhanced (Ryen and Kahn, 1975). Third, avoidance behavioral tendencies are automatically entailed by outgroup members (Carnaghi and Maass, 2006; Paladino and Castelli, 2008; Bianchi et al., 2018) which dampens the likelihood of establishing intergroup physical contact. Finally, although avoidance-like behaviors are triggered by a huge variety of outgroups, some social categories are stronger than others in discouraging physical contact, as in the case of those outgroups that raise fear of pathogen transmission (Neuberg et al., 2011; Golec de Zavala et al., 2014). Therefore, given the general reluctance to engage in physical contact with outgroup members and given the limited opportunity of establishing intergroup physical encounters, the beneficial effects of direct intergroup physical contact on prejudice can be impoverished.

To overcome these limitations, we suggest the use of physical contact within indirect forms of intergroup contact. It is worth noting that we do not argue in favor of indirect rather than direct forms of physical intergroup contact as a tool to ameliorate intergroup relations, rather we suggest that indirect forms of intergroup physical contact might be widely applicable in different cultural contexts. Indeed, indirect contact strategies do not imply direct interactions between groups, and are based on less intrusive approaches. One of the most effective indirect contact strategies is imagined contact (e.g., Crisp et al., 2010), in which participants are asked to imagine themselves interacting with an outgroup member. Although this manipulation implies a simulated scene, rather than an actual intergroup interaction, its positive effects on outgroup evaluation have been consistently observed on a variety of outgroup targets (Miles and Crisp, 2014) both on explicit (e.g., Turner et al., 2007) and implicit attitudes (Turner and Crisp, 2010). Also, indirect forms of contact such as the imagined contact, might be very important as they may actually prepare people for future contact with outgroup members (e.g., Choma et al., 2014).

Research on imagined contact has also stressed that mental imagery allows the activation of mental structures which could trigger responses associated with real experiences (see Crisp et al., 2009). Specifically, Turner et al. (2007) suggest that the mental simulation of an interaction with an outgroup member may lead participants to trigger processes which resemble the ones involved in real contact situations.

Beyond findings regarding the intergroup context, research carried out with fMRI (functional magnetic resonance imaging) and PET (i.e., Positron emission tomography) has demonstrated that mental imagery recruits similar brain networks as those involved in perception, memory, emotion, and motor control (Kosslyn et al., 2001). As far as touch is concerned, Lucas et al. (2015) compared the brain regions recruited during actual and imagined touch. Results indicated that the anterior insula is involved both when experiencing and also imagining touch (i.e., sensation of the touch), suggesting that imagined physical contact could, at least in part, resemble direct physical contact.

Notwithstanding these promising results showing an overlap between actual and imagined physical contact, few studies have recast the analyses of imagined physical contact within intergroup contexts. However, these studies (Choma et al., 2014; Hodson et al., 2015) provide only indirect support to the positive relationship between imagined intergroup physical contact and outgroup attitudes. Hodson et al. (2015) asked participants to imagine an elaborated contact with a homeless person, in which physical contact, cooperation and trust building exercises were encouraged (vs. a neutral, control scenario). An elaborated contact requires participants to imagine an interaction with an outgroup member, and implements the imagery by asking them to provide details of the intergroup encounter (see Husnu and Crisp, 2010). Compared to the control condition, the elaborated imagined contact weakened the relationship between disgust sensitivity and prejudice toward homeless individuals. In similar vein, Choma et al. (2014) after having assessed participants’ prejudice, asked participants to imagine a physical encounter with an outgroup member. Imagined physical contact was introduced in the form of team-building exercises (i.e., “thumb war” session), which again, did not only require a physical contact with an outgroup member but also a cooperative interaction to solve the task. Participants’ prejudice was then assessed again, before and after an actual team-building session (i.e., wrist loops) which required cooperation with an outgroup member and served as a cover story to establish a real physical contact. Results indicated that participants reported significantly reduced prejudice after the elaborated imagined physical contact session and these positive attitudes remained stable across the following sessions.

Although these studies suggest that imagined physical contact with an outgroup member may be effective in improving intergroup relations, the experimental manipulation described above did not only involve an imagined intergroup physical contact, but also included other variables, such as cooperation which has shown to improve *per se* intergroup attitude (Gaertner et al., 1990). Specifically, Gaertner and colleagues found that cooperation leads participants to perceive individuals as being part of one common group and also leads to reduced intergroup bias, and suggested that intergroup cooperation decreases intergroup bias because it modifies participants’ representation of two groups to one larger group. Hence, the specific contribution of imagined physical contact on the outcome variables still remains unclear. The current set of studies intends to fill this gap, by gaining a deeper understanding of whether and how imagined intergroup physical contact *per se* might improve intergroup attitudes. As previous evidence on this issue has mainly relied on explicit measures (Studies 1 and 2 of the current paper), we sought to understand whether imagined intergroup physical contact could be effective in ameliorating implicit intergroup attitudes (Study 3). It is worth noting that only few studies on imagined intergroup contact have relied on implicit measures so far (Turner and Crisp, 2010; Vezzali et al., 2012). Hence, this study further contributes to the understanding of how particular forms of imagined contact may affect not only explicit but also implicit attitudes.

Furthermore, we intend to ascertain whether it is the experience of imagining a physical contact *per se* that promotes harmonious intergroup relations or it is the specific imagined experience of a physical contact with an outgroup member that translates in improved intergroup attitudes (Studies 2 and 3). Importantly, this research question has not been addressed yet by both studies on actual and imagined physical intergroup encounters (Choma et al., 2014; Seger et al., 2014; Hodson et al., 2015). This aim is of particular significance as it starts specifying the psychological mechanisms entailed by the imagined intergroup physical encounters, since it specifies whether the generalization of the information gathered in the imagined physical encounter to the group as a whole occurs only when the (out)group membership of the encountered individual is salient (Rothbart and John, 1985; Hewstone and Brown, 1986). Additional information on the psychological processes involved in the imagined intergroup physical contact is provided by Study 3, in which participants were either primed with a physical intergroup encounter or imagined the same physical intergroup encounter. In so doing, we were able to test whether it is the perceptual experience or the mental simulation of an intergroup physical contact that exerts its positive impact on intergroup attitudes.

## Study 1

In Study 1, we question whether specifically imagining a physical contact with an outgroup member (i.e., an immigrant) compared to a standard control condition (i.e., an outdoor scene) reduces intergroup bias. This would allow gathering initial evidence on the effectiveness of imagined intergroup physical contact on intergroup attitudes. To address this aim we take advantage of the elaborated imagined contact paradigm. Elaborated imagined contact allows participants to imagine a more vivid, contextually situated intergroup contact scenario, contributing to the creation of a more detailed behavioral script which has shown to enhance the positive effects of imagined contact on intergroup attitudes (Crisp et al., 2010; Husnu and Crisp, 2010).

We implement this paradigm by introducing printed pictures of a physical encounter. With respect to the traditional paradigm employed in imagined contact research, we reckon that a visual cue could guide participants in the situation and help create a more vivid idea of the physical encounter, giving rise to a detailed form of elaborated imagined contact. This endeavor is guided by research demonstrating that a vivid imagery process is found after prompting participants with a concrete picture of the to-be-imagined content compared to a condition in which no picture is administered, and this leads to a more positive attitude toward the content (Babin and Burns, 1997). Also, providing participants with visual cues together with imaginary instructions facilitates individuals to create a vivid mental imagery (Finke, 1989; Ram et al., 2007).

The choice of providing participants with a specific form of physical contact, displayed by the visual cue (i.e., picture of a hand touch), rather than asking them to merely imagine a physical contact, is guided by two different, albeit related reasons. First, this procedure would secure us to make clear the body parts involved in the contact. Specifically, we decided to rely on a picture portraying a hand touch as the contact involving these body parts is likely to be processed as not intrusive and is widely accepted also when administered by a stranger (Suvilehto et al., 2015). Second, relying on a picture of a hand touch cues the specific modality in which the physical contact occurs. Indeed, even when physical contact involves the same specific body parts, the modality in which the physical contact is established may convey different emotional meanings (Hertenstein et al., 2006a). Exposing participants to the same picture portraying the same modality of physical contact enhances the chance that mental imagery would be elaborated based on that modality.

### Materials and Methods

#### Participants

One hundred twenty-seven undergraduate students from a university in northern Italy voluntarily took part in the study. We decided *a priori* to rely on a sample of 120 participants. This decision was backed by a sensitivity analyses (α err. prob. = 0.05, Power [1-β err. prob.] = 0.8, *N* = 120) which indicated a minimal detectable effect (MDE) size f = 0.11. Hence, the smallest *real* effect size which we would be able to detect (at 80% power) with this sample size falls within the small-effect size area (Cohen, 1988).

As we *a priori* decided to not include in the experimental sample non-Italian participants that did not speak Italian as their first language, we collected additional participants to achieve the required N.

Moreover, and by computing the achieved power of the current study (f = [$\eta_{p}^{2}$/(1-$\eta_{p}^{2}$)]^0.5^ = 0.18, α err. prob. = 0.05, *N* = 122), we showed that Power [1-β err. prob.] = 0.99.

Participants who indicated to be non-Italian and who did not speak Italian as their first language (*n* = 5) were eliminated from analysis, leaving the final sample to *n* = 122^1^. Participants were *n* = 61 female and *n* = 60 male students, and *n* = 1 did not report the gender. Participants’ age ranged between 19 and 33 years (*M* = 22.94, *SD* = 2.89). Participants were randomly assigned to one of two experimental conditions, namely either to the imagined intergroup physical contact condition (i.e., InterPC; *n* = 62) or to the control condition (*n* = 60).

#### Procedure

Participants were provided a questionnaire purportedly concerning the way people imagine social situations. Participants reported their nationality and native language on the first page, thus making their national-group identity salient (for similar procedure, see Steele and Aronson, 1995). Subsequently, they were presented with a colored picture which was located in front of them. Participants in the intergroup physical contact condition (i.e., InterPC) saw a picture displaying two hands of two distinct persons. One hand was depicted on the left side and one hand was displayed on the right side. The hand on the right was portrayed palm-down on the back of the hand displayed on the left, thus portraying a hand touch. The hand displayed on the right side of the picture was the hand of a White individual, while the hand on the left side was the hand of a Black individual, thus portraying an intergroup hand touch.

In the InterPC condition participants read the following instruction displayed above the picture:

We ask you to look at this picture for 1 min and to identify with one of the two main characters. Specifically, we ask you to imagine that the hand depicted on the right side is yours and that it is touching the hand of an immigrant. Participants further read: “Imagine feeling at ease during this contact and imagine it to be a positive experience in which you discover unexpected things” (for similar procedures see Turner et al., 2007; Stathi and Crisp, 2008).

In the control condition participants saw a picture portraying an outdoor scene (for similar procedures, see Turner et al., 2007) and were asked to follow the instruction displayed above the picture:

“We ask you to look at this picture for 1 min and to identify yourself in that scene. Specifically, we ask you to imagine that you are outdoors, in the portrayed environment.” As in the InterPC condition, participants further read: “Imagine feeling at ease and imagine it to be a positive experience in which you discover unexpected things” (for similar procedures see Turner et al., 2007; Stathi and Crisp, 2008). In sum, participants saw one picture per condition which was presented to them only once. The fact that participants went on a single (and not multiple) imaginary trial is consistent with the experimental procedure outlined by research on imagined intergroup contact (Crisp et al., 2010).

Following this manipulation, like in experiments dealing with imagined contact, to reinforce the effect of the imagery task participants were asked to report all the feelings they had experienced and the thoughts that had come to their mind while imagining themselves in that situation. Participants were given up to 2 min to report their reactions and were then provided with the dependent measures. Specifically, we intended to measure participants’ intergroup bias. To attain this aim, participants read that our lab was about to organize a short study in conjunction with our department, and that data collected would be used to organize a future study which would have taken place the next month. Moreover, participants read that the study aimed at making Italians interact with other Italians, immigrants with other immigrants, or Italians with immigrants. As a cover story, participants subsequently read that we were interested in knowing whether they were willing to take part in the study. Participants were then requested, regardless of their willingness to actually participate in the study, to indicate with whom they would be happy to work, and in which of the following couples they wanted to be put into (for similar procedure, see Turner et al., 2007). In this respect, participants were asked to indicate their preference for being paired with another Italian and with an immigrant on a scale ranging from 1 ( = *not at all*) to 9 ( = *very much*). As a part of the cover story, the immigrant-immigrant couple was also mentioned and participants were asked to not consider the mentioned couple if they were Italians.

After completing the dependent measure, participants reported the gender and age; they were thanked and fully debriefed. This study was carried out in accordance with the recommendation of APA guidelines and the local Ethical Committee. All participants gave written informed consent in accordance with the Declaration of Helsinki.

### Results and Discussion

Participants’ ratings were analyzed by means of an ANOVA 2 (Condition: control vs. InterPC) × 2 (Target: Italian vs. immigrant), with the former factor as a between-participants variable and the latter factor as a within-participants variable. As participants’ gender did not impact on the interaction of interest, analyses were carried out leaving this factor aside.

**FIGURE 1:**
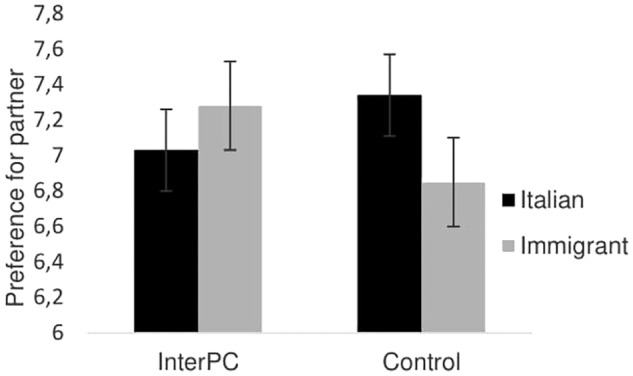
Preference for partner as a function of the experimental condition in Study 1. Error bars represent standard errors.

The analysis of variance revealed that no main effect of target was found, *F*(1,118) = 0.45, *p* = 0.51, $\eta_{p}^{2}$ = 0.004. The condition by target interaction turned out to be significant, *F*(1,118) = 4.03, *p* = 0.05, $\eta_{p}^{2}$ = 0.033, indicating that the relative preference for being paired with an Italian over an immigrant was lower in the InterPC (*M*_diff_. = −0.25, *SE* = 0.27) than in the control condition (*M*_diff_. = 0.49, *SE* = 0.25; see **Figure 1**).

These results provided initial support for the role of imagined intergroup physical contact in leveling intergroup bias. Participants who imagined to take the perspective of an ingroup member touching the hand of an outgroup member, labeled as an immigrant, showed lower levels of intergroup bias compared to those participants who were asked to imagine themselves in a positive outdoor scene.

The second study sought to replicate results found in Study 1, and started questioning whether the touch itself could account for a reduction of intergroup bias or whether touching an outgroup member, but not another individual, could likely be responsible for a reduction in intergroup bias responses.

## Study 2

In Study 2, we intended to investigate whether it is imagining a physical contact with an outgroup member (i.e., an immigrant) rather than an individual (i.e., a person) the basis for intergroup bias reduction. This study is particularly informative on the generative mechanism at the basis of the expected effects as it would clarify that it is not a physical contact *per se*, but that a specific physical contact with an outgroup member is needed to improve intergroup attitudes.

### Materials and Methods

#### Participants

One hundred twenty-eight undergraduate students from a university in northern Italy voluntarily took part in the study. As the experimental design of Study 2 was similar to the experimental design of Study 1, the same N rule was adopted in this study. Moreover, and by computing the achieved power of the current study (f [$\eta_{p}^{2}$/(1-$\eta_{p}^{2}$)]^0.5^ = 0.19, α err. prob. = 0.05, *N* = 122), we showed that Power [1-β err. prob.] = 0.99.

Participants who stated to be non-Italian and who did not speak Italian as their first language (*n* = 6) were eliminated from analysis, leaving the final sample to *n* = 122^1^. Participants were *n* = 67 female and *n* = 55 male students whose age ranged between 18 and 33 years (*M* = 22.67, *SD* = 3.15). Participants were randomly assigned to one of two experimental conditions: IntraPC (*n* = 59) and InterPC (*n* = 63).

#### Procedure

The experimental procedure was the same as in Study 1, otherwise specified. Participants were exposed to a picture of one hand touching another hand. In the InterPC condition, participants saw a hand of a White individual touching the hand of Black individual, which had been labeled as an immigrant as in Study 1. In the intragroup physical contact condition (i.e., IntraPC), participants saw the same hand of a White individual as in the InterPC, but in this case touching the hand of another White individual. In the IntraPC condition participants received exactly the same instruction as participants in the InterPC, except that the term “immigrant” was replaced by the term “person.” The dependent variable was the same as in Study 1.

After completing the dependent measure, participants reported their gender and age. Finally, they were thanked and fully debriefed. This study was carried out in accordance with the recommendation of APA guidelines and the local Ethical Committee. All participants gave written informed consent in accordance with the Declaration of Helsinki.

### Results and Discussion

Participants’ ratings were analyzed by means of an ANOVA 2 (Condition: IntraPC vs. InterPC) × 2 (Target: Italian vs. immigrant), with the former factor as a between-participants variable and the latter factor as a within-participants variable. As participants’ gender did not interact with any variable, analyses were carried out leaving this factor aside.

**FIGURE 2:**
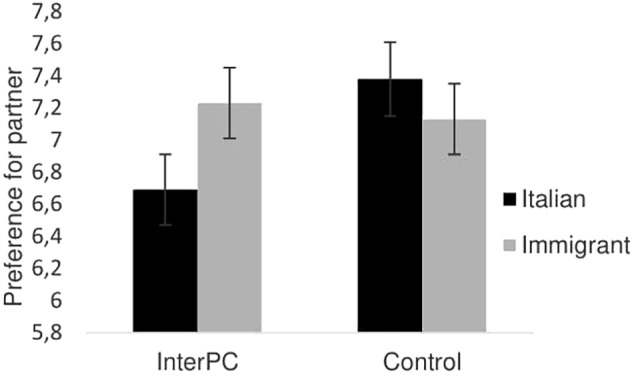
Preference for partner as a function of the experimental condition in Study 2. Error bars represent standard errors.

The analysis of variance revealed that no main effect of target was found, *F*(1,115) = 0.58, *p* = 0.45, $\eta_{p}^{2}$ = 0.005. The condition by target interaction turned out to be significant, *F*(1,115) = 4.26, *p* = 0.04, $\eta_{p}^{2}$ = 0.036, showing that the relative preference for being paired with an Italian over an immigrant was lower in the InterPC (*M*_diff_. = −0.54, *SE* = 0.29) than in the IntraPC (*M*_diff_. = 0.25, *SE* = 0.25) condition (see **Figure 2**).

This pattern of results corroborated our hypothesis that imagined intergroup physical contact worked as an intergroup bias reduction device. Participants who imagined to take the perspective of an ingroup member touching the hand of an outgroup member, labeled as an immigrant, showed lower levels of intergroup bias compared to those participants who took the perspective of an ingroup member touching the hand of another individual. Importantly, this study further clarifies the generative mechanism at the basis of the observed results. Indeed, it is not imagining taking the perspective of an ingroup member touching anyone that leads to a reduction in intergroup bias. By contrast, our results pointed that the imagined physical interaction, molded on the visual cue, should specifically involve an outgroup member to be effective in leveling intergroup bias.

#### Cross-Experimental Analyses

The participant sample of Studies 1 and 2 came from the same pool. Also, the procedure and the stimuli were the same, except for the control conditions, being these an outdoor scene in Study 1 and an imagined physical contact involving a White individual in Study 2. The sample sizes of the experimental samples were almost identical. Data collection occurred in an overlapping time period. Hence, the two studies were homogenous. Statistical cross-examination of these studies could be theoretically reliable (for a similar rationale and procedure, see Cherubini et al., 2013). The cross-experimental analyses would inform us about whether results in Studies 1 and 2 were (a) stable across studies as far as the InterPC conditions were concerned and (b) independent of the type of controls.

Data from these studies were merged together, and analyzed by using the type of study (i.e., Study 1 vs. Study 2) as a between-participants factor. Specifically, participants’ ratings were analyzed by means of an ANOVA 2 (type of study: Study 1 vs. Study 2) × 2 (Condition: controls vs. InterPC) × 2 (Target: Italian vs. immigrant), with the former two factors as between-participants variables and the latter factor as a within-participants variable. Results indicated a significant condition by target interaction, *F*(1,233) = 8.29, *p* = 0.004, $\eta_{p}^{2}$ = 0.034. Inspection of the means revealed that the relative preference for being paired with an Italian over an immigrant was lower in the InterPC (*M*_diff_. = −0.39, *SE* = 0.20) than in the control (*M*_diff_. = 0.37, *SE* = 0.18) conditions. Importantly, the condition by target interaction was not further qualified by the type of study, *F*(1,233) = 0.010, *p* = 0.92, $\eta_{p}^{2}$ = 0.001. No other significant effects were found (*Fs* < 1.02, *ps* > 0.31).

**FIGURE 3:**
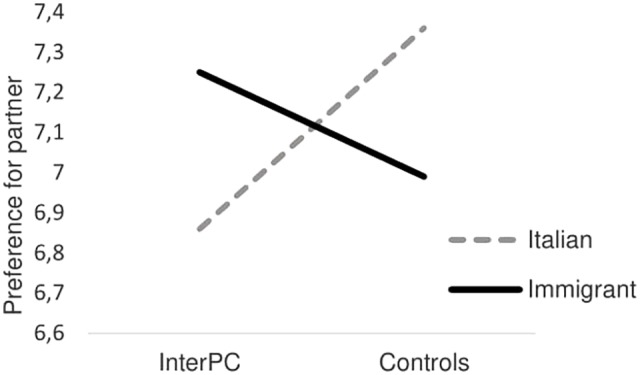
Preference for partner as a function of the experimental condition in the cross-experimental analyses.

This pattern of results corroborates our hypothesis about the effectiveness of the InterPC in leveling participants’ intergroup bias. The cross-experimental analyses confirmed that this effect was similar across studies. Indeed, intergroup bias was reduced in the InterPC compared to the control condition, regardless of the type of controls. It is worth noting that intergroup bias, being this a relative measure, has been operationalized as the preference for the ingroup over the outgroup. However, and to gather more information about the cognitive and motivational processes entailed by our manipulation, we further inspected the interaction by relying on pairwise comparisons. We performed these analyses on the Cross-experimental analyses because no significant differences occurred between studies on the main dependent variable. In so doing we could gather a more reliable conclusion on whether InterPC compared to the control condition enhanced preference for the outgroup or weakened the preference for the ingroup. Participants in the control conditions preferred working with an Italian rather than an immigrant person (*p* = 0.05), while participants’ preference for working with immigrants was higher compared to working with Italians in the InterPC condition (*p* = 0.03). Also, in the InterPC condition preference for working with Italians was lower compared to the control conditions (*p* = 0.03), while no difference occurred between conditions in terms of preference when considering immigrants (*p* = 0.26). Hence, it seems that imagined intergroup physical contact, compared to the control conditions, reduced ingroup bias (i.e., preference for the ingroup) rather than decreased rejection toward the outgroup (see **Figure 3**).

It is worth noting that ingroup preference and outgroup derogation may represent two distinct, albeit not necessarily independent, processes. Indeed, and in line with Allport’s (1954, see ‘Ingroup Formation’) claims, ingroup favoritism comes prior to the development of attitudes toward outgroups, and ingroup bias does not necessary imply negativity toward the outgroup. Indeed, ingroup bias could coexist with different types of attitudes toward the outgroup, including indifference and disdain (Brewer and Campbell, 1976; Brewer, 1999). Also, participants are typically reluctant to differentiate the ingroup from the outgroup on negative outcomes (outgroup derogation or rejection) while they are more willing to achieve intergroup differentiation on positive outcomes, thus testifying to a primacy of ingroup bias over outgroup derogation (i.e., positive-negative asymmetry; Mummendey et al., 1992). This pattern of behaviors typically occurs in non-conflictual intergroup contexts and in intimate intergroup contact (Duckitt and Mphuthing, 1998; Brewer, 1999). Evolutionary-based research also corroborates the idea that formation of group bonds (e.g., cooperation) and ingroup bias are often and primarily characterized by partiality toward the ingroup rather than hostility toward the outgroup (Fu et al., 2012).

As far as intergroup contexts are concerned, especially intimate intergroup encounters provide perceivers insights not only about the outgroup but also about the ingroup. Indeed, having intimate contact with members of national outgroups, leads ingroup members to display less ingroup attachment and ingroup pride (Pettigrew, 1997). This pattern of results is consistent with the ‘deprovincialization’ process (Pettigrew, 1998) which claims that intergroup contact can lead ingroup members to reappraise, namely questioning, the ingroup as the default standard and to distance from the ingroup (Verkuyten et al., 2010). Recasting the InterPC condition within this theoretical frame, InterPC represents an intimate contact with outgroup members, as it evokes a non-conflicting intergroup setting, and leads participants to establish a close form of intergroup contact based on communality (Seger et al., 2014). Hence, it might be plausible that these characteristics of the InterPC might activate a ‘deprovincialization’ process that maps onto an enhanced distancing from the ingroup (or a reduction of ingroup bias), which is observed in a lower probability of selecting ingroup members in the InterPC than in the control conditions. This line of reasoning is consistent with recent research endeavors that have found support for the idea that ingroup distancing may underlie part of the intergroup contact effects (Pettigrew, 2009; Tausch et al., 2010; Kauff et al., 2016).

## Study 3

In Study 3, we aimed to further understand whether the effects of the intergroup physical contact manipulation could extend beyond explicit attitudes and affect also implicit attitudes. In Study 3, participants were exposed to the same pictures as in Study 2, depicting a white hand touching another white hand (i.e., IntraPC) or a white hand touching the hand of a Black individual labeled as an immigrant (i.e., InterPC). Additionally, a third experimental condition was here added. Participants in this condition were exposed to the same picture used in the InterPC condition, but in this case they were asked to evaluate the quality of the picture and therefore did not engage in the imagination task (i.e., InterPC-quality). We reasoned that being exposed to a picture of an intergroup physical contact without imagining oneself in the depicted situation (i.e., InterPC-quality) would not lead to a consistent level of prejudice reduction as in the InterPC. If this were the case, we put forward that a visual cue portraying an intergroup physical contact would become effective only when used as the basis for the imagery process.

In Study 3, we relied on an implicit measure of attitudes, the paper-and-pencil Implicit Association Test (IAT; Schwartz et al., 2003), to tap participants’ spontaneous outgroup attitudes (i.e., prejudice and stereotypical prejudice). The IAT measures the automatic association of positive and negative attributes and concepts (i.e., Italians and immigrants in this case), in other words, the presence/absence of an automatic preference for one group over another (e.g., Newheiser and Olson, 2012). For this reason, we reckoned that this measure could represent a useful tool to assess intergroup bias from an implicit point of view, extending the results obtained in Studies 1 and 2.

On the basis of results of Studies 1 and 2, we hypothesized that, compared to participants in the InterPC, participants in the IntraPC would display a less positive/more stereotypical attitude, thus extending the effectiveness of InterPC to implicit measures (Hypothesis 1). Second, although participants were exposed to the same pictorial stimulus displaying an intergroup touch both in the InterPC and InterPC-quality condition, we expected participants in the InterPC condition to show reduced levels of implicit prejudice/stereotype than participants in the InterPC-quality condition, thus testifying to the key role played by mental imagery over the mere pictorial intergroup touch in reducing outgroup prejudice (Hypothesis 2). In sum, we put forward that participants in the interPC would display a more positive/less stereotyped attitude toward the outgroup than in both the IntraPC and InterPC-quality condition, while no difference should occur between the IntraPC and InterPC-quality condition (Hypothesis 3).

### Materials and Methods

#### Participants

One hundred thirty-eight undergraduate students from a university in northern Italy took part in the study in exchange for course credits during a social psychology class. Data were gathered in a collective session in class. The total number of students enrolled in the first year was around 150, with class attendance estimated around 80%. As we could not anticipate the number of participants that would attend the class on the day in which the experiment was scheduled, we were not able to anticipate the exact number of participants albeit, based on prior class attendance, we estimated to collect at least *N* = 120 participants. A sensitivity analyses (α err. prob. = 0.05, Power [1-β err. prob.] = 0.8, *N* = 120) indicated an MDE equal to f = 0.29. Hence, the sample size is adequate to detect a real effect size that falls within the small to intermediate effect size area (Cohen, 1988). The final sample approximated the required N.

Moreover, and by computing the achieved power of the current study (*d* = 0.403, α err. prob. = 0.05, *N* = 133), we showed that Power [1-β err. prob.] = 0.71.

Participants who stated to be non-Italian and who did not speak Italian as their first language (*n* = 2) were eliminated from analysis. In addition, three other participants were eliminated from analysis due to an error rate on the IAT greater than 3.5 *SD*, leaving the total sample to *n* = 133^1^.

Participants were *n* = 96 female and *n* = 37 male students whose age ranged between 18 and 54 years (*M* = 20.53, *SD* = 3.30). Participants were randomly assigned to one of three experimental conditions: InterPC-quality (*n* = 36), IntraPC (*n* = 51), and InterPC (*n* = 46).

#### Procedure

Data were collected during a collective session in class. The same procedure used in Studies 1 and 2 was adopted. Participants answered the questions on nationality and native language as in Studies 1 and 2. Participants in the InterPC-quality and in the InterPC conditions were exposed to exactly the same picture. Specifically, in these two conditions, the hand on the left side was the hand of a Black individual. In sharp contrast, in the IntraPC condition the hand on the left side belonged to a White individual, as in Study 2.

In the InterPC-quality condition participants read the following instruction displayed above the picture: “We ask you to look at this picture for 1 min and evaluate whether the picture is out-of-focus, overexposed, at high-resolution, grainy. In other words, we ask you to evaluate the photographic quality of the image.” In the InterPC and IntraPC conditions participants received the same instructions used in the previous studies.

In sum, participants in the InterPC-quality as well as in the InterPC condition were exposed to exactly the same visual information cueing an intergroup physical contact. However, these two conditions differed in terms of imagery task which was present in the InterPC condition but not in the InterPC-quality condition.

Following this manipulation, participants in the InterPC-quality condition reported their judgment on the quality of the picture while participants in the IntraPC and InterPC were asked to report all the feelings they had experienced and the thoughts that had come to their mind while imagining themselves in that situation. Participants were given up to 2 min to report their reactions and were subsequently provided with the dependent measures.

**FIGURE 4:**
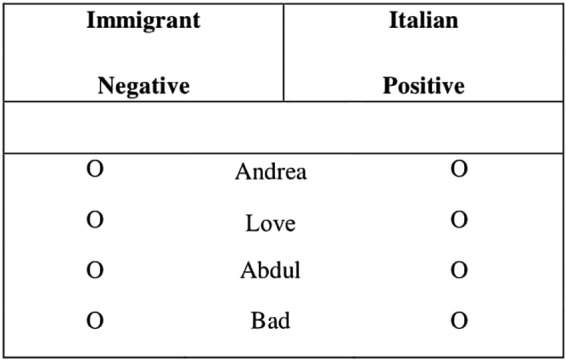
Example of the prejudice-IAT used in Study 3.

Participants’ prejudice and stereotypical prejudice were evaluated by means of two independent paper-and-pencil IATs (see Lowery et al., 2001; Schwartz et al., 2003). These measures were administered to all participants in the InterPC, IntraPC, and InterPC-quality conditions. In the prejudice-IAT, participants had to classify a list of words that fit into two categories (i.e., “Italians” and “Immigrants”) and two attributes (“positive” and “negative”). The categories and the attributes were presented on the top left and top right of the paper. On one sheet, the category ‘immigrant’ was paired with the attribute ‘negative’ on the upper left side of the paper, and the category ‘Italian’ was paired with the attribute ‘positive’ on the upper right side (see **Figure 4**). We herewith refer to this category-attribute combination as congruent block. Participants were presented with two separate columns of stimuli and they were asked to mark a sign to the left or to the right of each stimulus to indicate its appropriate category or attribute. The stimuli items included: Italian proper names, non-Italian proper names; positive words and negative words (see Greenwald et al., 1998 and **Table 1** for an example of the list of words used). For example, in the congruent condition, the to-be-classified word ‘Andrea’ should be check marked on the right, thus indicating its correct categorization as ‘Italian.’ If the to-be-classified word was check marked on the left side, this indicated an incorrect categorization of that word. On the next sheet the pairing was switched so that the category ‘immigrant’ was paired with the attribute ‘positive’ on the upper left side, and the category ‘Italian’ was paired with the attribute ‘negative’ on the upper right side. We herewith refer to this category-attribute combination as incongruent block. The order of presentation of the congruent and incongruent block was counterbalanced across participants.

**Table 1:** Example of stimuli used in the Prejudice (Positive vs. Negative) and in the Stereotypical-prejudice (Justice vs. Crime)-IAT.

Italian names	Immigrant names	Positive	Negative	Justice	Crime
Andrea	Hassad	Stupendo (Wonderful)	Male (Bad)	Onestà (Honesty)	Omicidio (Murder)
Antonio	Goran	Amore (Love)	Odio (Hate)	Legalità (Legality)	Stupro (Rape)
Mario	Abdul	Gioia (Joy)	Vomito (Vomit)	Lecito (Lecit)	Furto (Theft)

*English translation of the original stimuli is provided in brackets.*

The same procedure was adopted to measure participants’ stereotypical prejudice. In this case, the categories were again “Italian” and “Immigrant,” and, differently from the previously described IAT, the two attributes were “crime” and “justice”. The list of the to-be-categorized words were adjusted accordingly (Marchese and Milazzo, 2002).

Participants were given 20 s to classify the words in both the congruent (e.g., Immigrant+negative) and incongruent versions of each IAT (e.g., Immigrant+justice). The order of presentation of the prejudice- and the stereotypical-prejudice-IAT was counterbalanced among participants. Moreover, and for each type of IAT, the congruent and the incongruent versions were counterbalanced among participants. Participants then reported their age. Finally, they were thanked and fully debriefed. This study was carried out in accordance with the recommendation of APA guidelines and the local Ethical Committee. All participants gave written informed consent in accordance with the Declaration of Helsinki.

### Results and Discussion

#### Prejudice-IAT

The number of correct categorizations in one block compared to the other is the measure of relative association strength. Close associations (i.e., congruent block) between the category and the attribute that share the same response localization should make the task easier and the performance should be better (i.e., more correct categorizations) compared to the incongruent block. Specifically, in the prejudice-IAT, the (prejudice) congruent block indicated the extent to which (i) Immigrant names were more accurately associated with negative than positive attributes and (ii) Italian names were more accurately associated with positive than negative attributes. Conversely, the prejudice incongruent block indicated the extent to which (i) Immigrant names were more accurately associated with positive than negative attributes and ii) Italian names were more accurately associated with negative than positive attributes. Prejudice was here operationalized by subtracting the accuracy of incongruent associations from congruent associations.

As participants’ gender did not interact with the condition, analyses were carried out leaving this factor aside.

**FIGURE 5:**
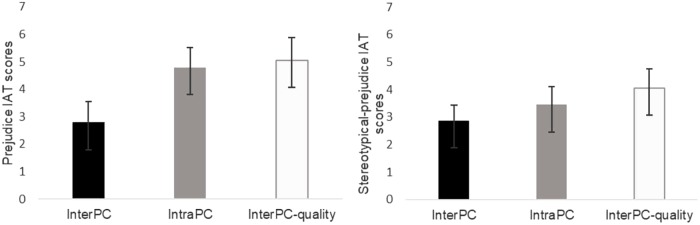
Prejudice and stereotypical-prejudice IAT scores as a function of the experimental condition in Study 3. Error bars represent standard errors.

Based on previous results, we carried out a planned contrast comparing InterPC (contrast weight: +1) to IntraPC (contrast weight: −1), and InterPC-quality (contrast weight: 0). This contrast allowed us to test whether intergroup bias was lower in the InterPC compared to the IntraPC condition, as previously demonstrated. This contrast was significant, *t*(130) = 1.95, *p* = 0.05. Also, we directly test whether InterPC lowered intergroup bias to a greater extent than the InterPC-quality. Therefore, we tested InterPC (contrast weight: +1) to IntraPC (contrast weight: 0), and InterPC-quality (contrast weight: −1), and this contrast was significant, *t*(130) = 2.01, *p* = 0.05. Also, the level of intergroup bias was very similar in the IntraPC and InterPC-quality condition, as demonstrated by the planned contrast comparing InterPC (contrast weight: 0) to IntraPC (contrast weight: −1), and InterPC-quality (contrast weight: +1), which was not significant, *t*(130) = 0.23, *p* = 0.82. In addition, the contrast InterPC (contrast weight: +2), IntraPC (contrast weight: −1) and InterPC-quality (contrast weight: −1) turned out to be significant *t*(130) = 2.30, *p* = 0.02 (see **Figure 5**).

These results confirmed the effectiveness of the InterPC in leveling intergroup bias over the two other conditions, which did not differ from each other.

#### Stereotypical-Prejudice-IAT

In the stereotypical-prejudice-IAT, the congruent block indicated the extent to which (i) Immigrant names were more accurately associated with words regarding crime rather than justice and (ii) Italian names were more accurately associated with words regarding justice rather than crime. Conversely, the incongruent condition indicated the extent to which (i) Immigrant names were more accurately associated with words regarding justice rather than crime and (ii) Italian names were more accurately associated with words regarding crime rather than justice. Similarly, to the prejudice-IAT, stereotyping was here operationalized by subtracting the accuracy of incongruent associations from congruent associations.

We performed the same contrast analysis as for the prejudice-IAT. We performed a planned contrast comparing InterPC (contrast weight: +1) to IntraPC (contrast weight: −1), and InterPC-quality (contrast weight: 0), and this contrast was not significant, *t*(130) = 0.67, *p* = 0.50. We then tested InterPC (contrast weight: +1) to IntraPC (contrast weight: 0), and InterPC-quality (contrast weight: −1), and this contrast was not significant, *t*(130) = 1.25, *p* = 0.21. Also, the planned contrast comparing InterPC (contrast weight: 0) to IntraPC (contrast weight: −1), and InterPC-quality (contrast weight: +1), was not significant, *t*(130) = 0.65, *p* = 0.52. The contrast InterPC (contrast weight: +2), IntraPC (contrast weight: −1) and InterPC-quality (contrast weight: −1) was not significant *t*(130) = 1.13, *p* = 0.26 (see **Figure 5**). These results indicated that the levels of implicit stereotypical prejudice were unaffected by the experimental manipulation.

Results of Study 3 confirm and extend the results of Study 2 to implicit measures, as InterPC compared to IntraPC reduced implicit prejudice toward the outgroup, confirming hypothesis 1. Therefore, results of Study 3 further corroborate the idea that it is not physical contact *per se* that promotes prejudice reduction, but that the physical encounter should involve an outgroup member in order to trigger prejudice reduction. Furthermore, results of Study 3 clarify the boundaries conditions of the imagined intergroup physical contact manipulation. InterPC leveled implicit outgroup prejudice only when participants were asked to imagine themselves in the intergroup physical contact interaction. Indeed, when participants were asked to merely process the perceptual features of the picture as in the InterPC-quality condition, they did not show consistent prejudice reduction, confirming hypothesis 2.

Interestingly, the InterPC affected participants’ implicit prejudice but not participants’ implicit stereotypical prejudice. Indeed, no difference among conditions was found on the stereotypical-prejudice-IAT (for a discussion of this null effect, see below).

## General Discussion

In three studies we analyzed the role played by imagined physical contact in ameliorating intergroup attitudes. Built upon the cross-over of studies showing that imagined contact, on the one hand, and actual physical contact on the other hand, can reduce outgroup prejudice and improve intergroup perception, we put forward that the mental imagery involving an intergroup physical contact may be a promising psychological device to lead perceivers to display more positive intergroup attitudes. To test this hypothesis, we tested whether seeing an image of an intergroup touch while imagining touching the hand of an outgroup member (i.e., an immigrant) improved intergroup attitudes.

Results of Study 1 indicate that imagining a physical contact with an outgroup member (i.e., an immigrant) compared to a control condition (i.e., an outdoor scene) reduced intergroup bias. These results allowed us to gather initial evidence on the effectiveness of imagined intergroup physical contact on intergroup attitudes. Although Study 1 answers to our initial question of whether imagined physical contact could improve outgroup attitudes, Study 1 does not allow to clearly understand whether it is a touch in general or an intergroup touch in particular which triggers a more positive intergroup attitude. Study 2, specifically addresses this question and provides information in this respect. By comparing an intragroup physical contact condition (i.e., IntraPC) and an intergroup physical contact condition (i.e., InterPC) we were able to demonstrate that it is not an imagined physical contact *per se*, but that specific imagined physical contact with an outgroup member is needed to ultimately improve intergroup attitudes.

In sum, and corroborated by the cross-experimental analyses, results of Studies 1 and 2 suggest that the positive imagined experience of touching an outgroup member specifically constitutes the grounds for generalizing the positive effects of the imagined encounter to the representation of the intergroup relation as a whole, independent of the type of control.

Furthermore, results from Study 3 show that the effects of imagined intergroup touch can be also detected at an implicit level. Indeed, participants in the InterPC condition showed lower levels of implicit prejudice compared to participants in the IntraPC condition, confirming the results found in Study 2. Study 3 sheds light on the crucial role played by mental imagery in ameliorating intergroup attitudes. Indeed, the exposure to a picture of an intergroup touch (i.e., InterPC-quality) did not produce the same positive outcomes as to when participants were also asked to imagine the intergroup touch (i.e., InterPC).

In sum, the combining results of the three studies suggest that imagined intergroup physical contact ameliorates intergroup attitudes both at explicit and implicit level. The fact that the imagined physical contact exerts its beneficial effects on overt and covert attitudes rules out the possibility that the observed effects were driven by demand characteristics, and suggests that imagined intergroup physical contact ameliorated both controlled and spontaneous attitudes.

Importantly, findings of Study 3 show that imagined intergroup physical contact ameliorates evaluative attitudes but does not alter group stereotyping. Two different reasons might account for these results. First, stereotypes refer to the semantic knowledge associated with the outgroup (Hamilton, 1981; Rumiati et al., 2014). Stereotype revision is typically triggered by the exposure to actual or imagined counterstereotypical exemplars (e.g., Dasgupta and Greenwald, 2001; Carnaghi and Yzerbyt, 2007). Since in the imagined intergroup physical contact perceivers are not exposed to any counterstereotypical instance, there is no reason for stereotype revision to occur. Second, prejudice is conceived as the affective response toward the outgroup (Amodio and Devine, 2006), and is found to be predictive of appetitive behaviors. Indeed, evidence shows that prejudice, but not stereotype about an outgroup, is associated with both avoidance tendency and physical distance from a member of that outgroup, thus proving a selective relation between prejudice, and aversive behaviors (Dovidio et al., 2002; Amodio and Devine, 2006). The imagined intergroup physical contact might be framed as induced appetitive behavior, since the perceiver is requested to be physically close to the outgroup member. Together, these claims suggest that imagined intergroup physical contact likely operates under affective rather than semantic learning mechanisms (Amodio and Lieberman, 2009), and opens up the question of whether intergroup physical contact additionally works through intergroup anxiety route to reduce outgroup prejudice.

Our findings extend and further clarify the results found by Choma et al. (2014) and Hodson et al. (2015) showing that imagined intergroup physical contact is *per se* effective in ameliorating intergroup attitudes even when participants are not provided with additional information concerning the type of interaction with the outgroup (e.g., intergroup cooperative setting). Also, this set of studies broadens the theoretical frame of imagined contact by showing that even specific forms of imagined contact, such as the one represented by a physical encounter, could be a promising strategy to improve intergroup relations. Note that this set of studies was not designed to compare the traditional form of imagined contact with imagined intergroup physical contact. Indeed, the primary aim of this research is to inform on the effects of physical contact also in intergroup contexts, a topic which has not yet been fully investigated in its indirect forms. In addition, most of the research has focused on the effects of direct physical contact in interpersonal settings while less attention has been given to the role played by physical contact in intergroup settings in both its direct and indirect forms. Thus, this research is informative in the sense that it expands the limited literature on the effects of intergroup physical contact on outgroup attitudes by using indirect contact strategies. Nevertheless, future studies could aim at comparing imagined intergroup physical contact and traditional forms of imagined contact to test if and in which contexts (e.g., countries in which touch is more frequently used or countries which do not often involve in physical contact), one could be more effective than the other.

There are also a number of limits which should be taken into account when evaluating the effectiveness of the imagined intergroup physical contact. First, despite the promising results found both on explicit and implicit attitude measures, we did not test the efficacy of the imagined intergroup physical contact over time. Indeed, we did not ascertain whether the predicted shift in intergroup attitudes was stable over time and resistant to subsequent negative information concerning the outgroup. Said otherwise, for an intervention to be useful in improving intergroup relations, immediate effects on intergroup attitudes should be acknowledged, but also longer lasting effects should be demonstrated (Dasgupta and Greenwald, 2001). Second, the impact of imagined intergroup physical contact on intergroup attitudes is small in terms of effect size. Despite the size of the effect, our findings are not trivial as they are the outcome of a brief and single imagined physical contact. It might be plausible that reiterated experiences of imagined intergroup physical contact could reach a larger effect. More studies are needed to truly identify the boundaries conditions that allow the imagined intergroup physical contact to be successful in changing outgroup attitudes.

## Author Contributions

SS and AC designed the research and wrote the article. SS and VP performed the research. SS, AC, and MG analyzed data. MB, VP, and MG provided critical revisions.

## Conflict of Interest Statement

The authors declare that the research was conducted in the absence of any commercial or financial relationships that could be construed as a potential conflict of interest.

## Footnotes

1 Among the sample no participant belonged to the African–Italian community.

## References

[B1] Allport G. W. (1954). *The Nature of Prejudice.* Cambridge, MA: Addison-Wesley.

[B2] Amodio D. Lieberman M. (2009). “Pictures in our heads: contributions of fMRI to the study of prejudice and stereotyping,” in *Handbook of Prejudice, Stereotyping, and Discrimination*, ed. Nelson T. (Hillsdale, NJ: Lawrence Erlbaum Associates), 347–366.

[B3] Amodio D. M. Devine P. G. (2006). Stereotyping and evaluation in implicit race bias: evidence for independent constructs and unique effects on behavior. *J. Pers. Soc. Psychol.* 91 652–661. 10.1037/0022-3514.91.4.652 17014291

[B4] Babin L. A. Burns A. C. (1997). Effects of print ad pictures and copy containing instructions to imagine on mental imagery that mediates attitudes. *J. Advert.* 26 33–44. 10.1080/00913367.1997.10673527

[B5] Bianchi M. Carnaghi A. Shamloo S. E. (2018). Intergroup attitudes accessibility and motor approach-avoidance responses in White and Black individuals in Portugal. *Psicol. Soc.* 2 148–164.

[B6] Brewer M. B. (1999). The psychology of prejudice: ingroup love and outgroup hate? *J. Soc. Issues* 55 429–444. 10.1111/0022-4537.00126

[B7] Brewer M. B. Campbell D. T. (1976). *Ethnocentrism and Intergroup Attitudes: East African Evidence.* New York, NY: Wiley.

[B8] Carnaghi A. Maass A. (2006). Effetti delle etichette denigratorie sulle risposte comportamentali. *Psicol. Soc.* 1 121–132.

[B9] Carnaghi A. Yzerbyt V. Y. (2007). Subtyping and social consensus: the role of the audience in the maintenance of stereotypic beliefs. *Eur. J. Soc. Psychol.* 37 902–922. 10.1002/ejsp.402

[B10] Cherubini P. Rusconi P. Russo S. Crippa F. (2013). Missing the dog that failed to bark in the nighttime: on the overestimation of occurrences over non-occurrences in hypothesis testing. *Psychol. Res.* 77 348–370. 10.1007/s00426-012-0430-3 22415224

[B11] Choma B. L. Charlesford J. J. Hodson G. (2014). Reducing prejudice with (Elaborated) Imagined and physical intergroup contact interventions. *Curr. Res. Soc. Psychol.* 22 20–26.

[B12] Cohen J. (1988). *Statistical Power Analysis for the Behavioral Sciences.* Hillsdale, NJ: Erlbaum.

[B13] Crisp R. J. Husnu S. Meleady R. Stathi S. Turner R. N. (2010). From imagery to intention: a dual route model of imagined contact effects. *Eur. Rev. Soc. Psychol.* 21 188–236. 10.1080/10463283.2010.543312

[B14] Crisp R. J. Stathi S. Turner R. N. Husnu S. (2009). Imagined intergroup contact: theory, paradigm and practice. *Soc. Personal. Psychol. Compass* 3 1–18. 10.1111/j.1751-9004.2008.00155.x

[B15] Dasgupta N. Greenwald A. G. (2001). On the malleability of automatic attitudes: combating automatic prejudice with images of admired and disliked individuals. *J. Pers. Soc. Psychol.* 81 800–814. 10.1037/0022-3514.81.5.800 11708558

[B16] Ditzen B. Neumann I. D. Bodenmann G. von Dawans B. Turner R. A. Ehlert U. (2007). Effects of different kinds of couple interaction on cortisol and heart rate responses to stress in women. *Psychoneuroendocrinology* 32 565–574. 10.1016/j.psyneuen.2007.03.011 17499441

[B17] Dovidio J. F. Esses V. M. Beach K. R. Gaertner S. L. (2002). “The role of affect in determining intergroup behavior: the case of willingness to engage in intergroup contact,” in *From Prejudice to Intergroup Emotions: Differentiated Reactions to Social Groups*, eds Mackie D. M. Smith E. R. (Philadelphia: Psychology Press), 153–171.

[B18] Duckitt J. Mphuthing T. (1998). Group identification and intergroup attitudes: a longitudinal analysis in South Africa. *J. Personal. Soc. Psychol.* 74 80–85. 10.1037/0022-3514.74.1.80 9457777

[B19] Erceau D. Guéguen N. (2007). Tactile contact and evaluation of the toucher. *J. Soc. Psychol.* 147 441–444. 10.3200/SOCP.147.4.441-444 17955753

[B20] Field T. (2001). *Touch.* Cambridge, MA: MIT Press.

[B21] Field T. Diego M. Hernandez-Reif M. (2007). Massage therapy research. *Dev. Rev.* 27 75–89. 10.1016/j.dr.2005.12.002

[B22] Finke R. A. (1989). *Principles of Mental Imagery.* Cambridge, MA: MIT Press.

[B23] Firebaugh G. Davis K. E. (1988). Trends in Antiblack Prejudice, 1972-1984: region and Cohort Effects. *American Journal of Sociology* 94 251–272. 10.1086/228991

[B24] Fiske A. P. (1992). The four elementary forms of sociality: framework for a unified theory of social relations. *Psychol. Rev.* 99 689–723. 10.1037/0033-295X.99.4.689 1454904

[B25] Fiske A. P. (2004). “Four modes of constituting relationships: consubstantial assimilation; space, magnitude, time, and force; concrete procedures; abstract symbolism,” in *Relational Models Theory: A Contemporary Overview*, ed. Haslam N. (Mahwah, NJ: Erlbaum), 61–146.

[B26] Fu F. Tarnita C. E. Christakis N. A. Wang L. Rand D. G. Nowak M. A. (2012). Evolution of in-group favoritism. *Sci. Rep.* 2:460. 10.1038/srep00460 22724059 PMC3380441

[B27] Gaertner S. L. Mann J. A. Dovidio J. F. Murrell A. J. Pomare M. (1990). How does cooperation reduce intergroup bias? *J. Personal. Soc. Psychol.* 59 692–704. 10.1037/0022-3514.59.4.692

[B28] Gallace A. Spence C. (2010). The science of interpersonal touch: an overview. *Neurosci. Biobehav. Rev.* 34 246–259. 10.1016/j.neubiorev.2008.10.004 18992276

[B29] Golec de Zavala A. Waldzus S. Cypryanska M. (2014). Prejudice towards gay men and a need for physical cleansing. *J. Exp. Soc. Psychol.* 54 1–10. 10.1016/j.jesp.2014.04.001

[B30] Greenwald A. G. McGhee D. E. Schwartz J. L. (1998). Measuring individual differences in implicit cognition: the implicit association test. *J. Personal. Soc. Psychol.* 74:1464 10.1037/0022-3514.74.6.1464 9654756

[B31] Guéguen N. Fischer-lokou J. (2003). Tactile contact and spontaneous help: an evaluation in a natural setting. *J. Soc. Psychol.* 143 785–787. 10.1080/00224540309600431 14658752

[B32] Hamilton D. L. (1981). “Stereotyping and intergroup behavior: some thoughts on the cognitive approach,” in *Cognitive Processes in Stereotyping and Intergroup Behavior*, ed. Hamilton D. L. (Hillsdale, NJ: Erlbaum), 333–353.

[B33] Hertenstein M. J. Holmes R. McCullough M. Keltner D. (2009). The communication of emotion via touch. *Emotion* 9 566–573. 10.1037/a0016108 19653781

[B34] Hertenstein M. J. Keltner D. App B. Bulleit B. A. Jaskolka A. R. (2006a). Touch communicates distinct emotions. *Emotion* 6 528–533. 10.1037/1528-3542.6.3.528 16938094

[B35] Hertenstein M. J. Verkamp J. M. Kerestes A. M. Holmes R. M. (2006b). The communicative functions of touch in humans, nonhuman primates, and rats: a review and synthesis of the empirical research. *Genet. Soc. Gen. Psychol. Monogr.* 132 5–94. 10.3200/MONO.132.1.5-94 17345871

[B36] Hewstone M. E. Brown R. E. (1986). *Contact and Conflict in Intergroup Encounters.* Basil: Blackwell.

[B37] Hodson G. Dube B. Choma B. L. (2015). Can (elaborated) imagined contact interventions reduce prejudice among those higher in intergroup disgust sensitivity (ITG-DS)? *J. Appl. Soc. Psychol.* 45 123–131. 10.1111/jasp.12281

[B38] Holt-Lunstad J. Birmingham W. A. Light K. C. (2008). Influence of a “Warm Touch” support enhancement intervention among married couples on ambulatory blood pressure, oxytocin, alpha amylase, and cortisol. *Psychosom. Med.* 70 976–985. 10.1097/PSY.0b013e318187aef7 18842740

[B39] Husnu S. Crisp R. J. (2010). Elaboration enhances the imagined contact effect. *J. Exp. Soc. Psychol.* 46 943–950. 10.1016/j.jesp.2010.05.014

[B40] Jakubiak B. K. Feeney B. C. (2016). Affectionate touch to promote relational, psychological, and physical well-being in adulthood: a theoretical model and review of the research. *Pers. Soc. Psychol. Rev.* 21 228–252. 10.1177/1088868316650307 27225036

[B41] Kauff M. Schmid K. Lolliot S. Al Ramiah A. Hewstone M. (2016). Intergroup contact effects via ingroup distancing among majority and minority groups: moderation by social dominance orientation. *PLoS One* 11:e0146895. 10.1371/journal.pone.0146895 26751203 PMC4709236

[B42] Kosslyn S. M. Ganis G. Thompson W. L. (2001). Neural foundations of imagery. *Nat. Rev. Neurosci.* 2 635–642. 10.1038/35090055 11533731

[B43] Lowery B. S. Hardin C. D. Sinclair S. (2001). Social influence effects on automatic racial prejudice. *J. Personal. Soc. Psychol.* 81:842. 10.1037/0022-3514.81.5.842 11708561

[B44] Lucas M. V. Anderson L. C. Bolling D. Z. Pelphrey K. A. Kaiser M. D. (2015). Dissociating the neural correlates of experiencing and imagining affective touch. *Cereb. Cortex* 25 2623–2630. 10.1093/cercor/bhu061 24700583 PMC4537425

[B45] Marchese M. Milazzo G. (2002). *L’agenda dei Telegiornali Sulle Notizie di Criminalità e Immigrazione: un Confronto fra il 2000 e il 2001.* Available at: http://www.osservatorio.it/download/criminalita.pdf

[B46] Miles E. Crisp R. J. (2014). A meta-analytic test of the imagined contact hypothesis. *Group Process. Intergroup Relat.* 17 3–26. 10.1177/1368430213510573

[B47] Montagu A. (1971). *Touching: The Human Significance of the Skin.* New York, NY: Columbia University Press.

[B48] Mummendey A. Simon B. Dietze C. Grünert M. Haeger G. Kessler S. (1992). Categorization is not enough: intergroup discrimination in negative outcome allocation. *J. Exp. Soc. Psychol.* 28 125–144. 10.1016/0022-1031(92)90035-I

[B49] National Center for Education Statistics [NCES] (2016). *U.S. Department of Education. Digest of Education Statistics, 2015 (NCES 2016-014).* Washington, D.C: National Center for Education Statistics.

[B50] Neuberg S. L. Kenrick D. T. Schaller M. (2011). Human threat management systems: self-protection and disease avoidance. *Neurosci. Biobehav. Rev.* 35 1042–1051. 10.1016/j.neubiorev.2010.08.011 20833199 PMC3024471

[B51] Newheiser A.-K. Olson K. R. (2012). White and black american children’s implicit intergroup bias. *J. Exp. Soc. Psychol.* 48 264–270. 10.1016/j.jesp.2011.08.011 22184478 PMC3241011

[B52] Paladino M.-P. Castelli L. (2008). On the immediate consequences of intergroup categorization: activation of approach and avoidance motor behavior toward ingroup and outgroup members. *Personal. Soc. Psychol. Bull.* 34 755–768. 10.1177/0146167208315155 18388255

[B53] Pettigrew T. F. (1997). Generalized intergroup contact effects on prejudice. *Personal. Soc. Psychol. Bull.* 23 173–185. 10.1177/0146167297232006

[B54] Pettigrew T. F. (1998). Intergroup contact theory. *Annu. Rev. Psychol.* 49 65–85. 10.1146/annurev.psych.49.1.65 15012467

[B55] Pettigrew T. F. (2009). Secondary transfer effect of contact: do intergroup contact effects spread to noncontacted outgroups? *Soc. Psychol.* 40:55 10.1027/1864-9335.40.2.55

[B56] Ram N. Riggs S. M. Skaling S. Landers D. M. McCullagh P. (2007). A comparison of modelling and imagery in the acquisition and retention of motor skills. *J. Sports Sci.* 25 587–597. 10.1080/02640410600947132 17365544

[B57] Rankin R. E. Campbell D. T. (1955). Galvanic skin response to Negro and white experimenters. *J. Abnorm. Soc. Psychol.* 51 30–33. 10.1037/h0041539 13242282

[B58] Remland M. S. Jones T. S. Brinkman H. (1995). Interpersonal distance, body orientation, and touch: effects of culture, gender, and age. *J. Soc. Psychol.* 135 281–297. 10.1080/00224545.1995.9713958 7650932

[B59] Rothbart M. John O. P. (1985). Social categorization and behavioral episodes: a cognitive analysis of the effects of intergroup contact. *J. Soc. Issues* 41 81–104. 10.1111/j.1540-4560.1985.tb01130.x

[B60] Rumiati R. I. Carnaghi A. Improta E. Diez A. L. Silveri M. C. (2014). Social groups have a representation of their own: clues from neuropsychology. *Cogn. Neurosci.* 5 85–96. 10.1080/17588928.2013.876981 24423240

[B61] Ryen A. H. Kahn A. (1975). Effects of intergroup orientation on group attitudes and proxemic behavior. *J. Pers. Soc. Psychol.* 31 302–310. 10.1037/h0076283

[B62] Schwartz M. B. Chambliss H. O. Brownell K. D. Blair S. N. Billington C. (2003). Weight bias among health professionals specializing in obesity. *Obes. Res.* 11 1033–1039. 10.1038/oby.2003.142 12972672

[B63] Seger C. R. Smith E. R. Percy E. J. Conrey F. R. (2014). Reach out and reduce prejudice: the impact of interpersonal touch on intergroup liking. *Basic Appl. Soc. Psychol.* 36 51–58. 10.1080/01973533.2013.856786

[B64] Stathi S. Crisp R. J. (2008). Imagining intergroup contact promotes projection to outgroups. *J. Exp. Soc. Psychol.* 44 943–957. 10.1016/j.jesp.2008.02.003

[B65] Steele C. M. Aronson J. (1995). Stereotype threat and the intellectual test performance of African Americans. *J. Pers. Soc. Psychol.* 69 797–811. 10.1037/0022-3514.69.5.797 7473032

[B66] Suvilehto J. T. Glerean E. Dunbar R. I. M. Hari R. Nummenmaa L. (2015). Topography of social touching depends on emotional bonds between humans. *Proc. Natl. Acad. Sci. U.S.A.* 112 13811–13816. 10.1073/pnas.1519231112 26504228 PMC4653180

[B67] Tausch N. Hewstone M. Kenworthy J. B. Psaltis C. Schmid K. Popan J. R. (2010). Secondary transfer effects of intergroup contact: alternative accounts and underlying processes. *J. Pers. Soc. Psychol.* 99 282–302. 10.1037/a0018553 20658844

[B68] Turner R. N. Crisp R. J. (2010). Imagining intergroup contact reduces implicit prejudice. *Br. J. Soc. Psychol.* 49 129–142. 10.1348/014466609X419901 19302731

[B69] Turner R. N. Crisp R. J. Lambert E. (2007). Imagining intergroup contact can improve intergroup attitudes. *Group Process. Intergroup Relat.* 10 427–441. 10.1177/1368430207081533

[B70] Verkuyten M. Thijs J. Bekhuis H. (2010). Intergroup contact and ingroup reappraisal: examining the deprovincialization thesis. *Soc. Psychol. Q.* 73 398–416. 10.1177/0190272510389015

[B71] Vezzali L. Capozza D. Giovannini D. Stathi S. (2012). Improving implicit and explicit intergroup attitudes using imagined contact: an experimental intervention with elementary school children. *Group Process. Intergroup Relat.* 15 203–212. 10.1177/1368430211424920

[B72] Vrana S. R. Rollock D. (1998). Physiological response to a minimal social encounter: effects of gender, ethnicity, and social context. *Psychophysiology* 35 462–469. 10.1111/1469-8986.3540462 9643061

